# Empiric mathematical model for predicting the content of alpha-acids in hop (*Humulus lupulus* L.) cv. Aurora

**DOI:** 10.1186/2193-1801-2-59

**Published:** 2013-02-19

**Authors:** Siniša Srečec, Barbara Čeh, Tanja Savić Ciler, Alenka Ferlež Rus

**Affiliations:** 1 Križevci College of Agriculture, M. Demerca 1, HR-48260 Križevci, Croatia; 2 Slovenian Institute of Hop Research and Brewing, Cesta Žalskega tabora 2, Žalec, SI-3310 Slovenia

**Keywords:** Hop, *Humulus lupulus* L, Alpha-acids, Accumulation of alpha-acids, Empiric mathematical model, Eurequa software

## Abstract

The aim of this research is to find a simple mathematical model due to sum of effective temperatures and rainfalls from second germination after spring pruning till the technological maturity of hop cones, in order to achieve reliable prognosis of alpha-acids content in hop cv. Aurora. After mathematical analyses of experimental data by Eurequa Formulize 0.96 Beta software 17 equations were offered, and after substituting the values of dependent and independent variables in all equations only one equation was chosen with p = 0.034 (p<0.05). This equation is not reliable in extremely drought year if crop evapotranspiration ET_0_ in July is higher than 4.5, primarily because of negative influence on formation and development of hop glandular trichomes. Considering achieved results it is possible to suggest following general equation for alpha-acids accumulation in hop: *y* = [(*k*_1_ *w*) − *k*_2_ − (*k*_3_ *w*^2^)/*x*] ÷ (−10) ↔ *ET*_0 *July*_ ≤ 4.5. Where *y* is alpha acids content in dry matter (%), *x* = sum of effective temperatures and *w* = sum of rainfals, both from second germination after spring pruning till technological maturity of hop cones. Coefficients *k*_*1*_, *k*_*2*_ and *k*_*3*_ are determined for cultivar Aurora (53.8, 453 and 1.33, respectively).

## Introduction

The alpha-acids are important quality parameter in the hop industry since their production significantly defines the global hop supply statistics (Pavlovic et al. 2008). The biosynthesis of hop secondary metabolites is divided into three biosynthetic pathways A, B and C, and biosynthesis of humulone or alpha-acids is the final step of C pathway (Nagel et al. 2008; Wang et al. 2008). Accumulation of alpha acids is most intensive in third and fourth week after hop flowering (Wang et al. 2008). Mozny et al. (2009) found a positive impact of rainfall and a negative effect of temperature on alpha-acids accumulation in Czech Saaz hop cultivar, as well as Srečec et al. 2008in Aurora hop cultivar. On the other hand, Kučera and Krofta (2009) found that the strongest impact on the alpha-acid content was exerted by air temperatures in July and rainfall had significant effects during the period from May to July, while in August the impact of a rainfall was negligible. Pavlovič et al. (2012) found that the impact of weather parameters on the alpha-acids in hops can be linked with the emergence of certain phases of recorded phenomena in the plants, which do not coincide in time with the dispensation of each month. Generative bodies in the cultivar Aurora begin to develop in the second half of June (week 26), and the plant is in full blooming by the mid July (week 28). These results stays in line with results of Srečec et al. (2008) who found significant decrease of alpha-acids accumulation if average daily reference crop evapotranspiration in June is higher or equal to 4.5. Srečec et al. (2008) on the basis of results of linear and multiple correlations suggested a following functional equation for accumulation of alpha acids (equation 1).1

where:

- ***A*** – content of alpha-acids in dry matter (%)
- ***T*** – sum of effective temperatures from second germination to hop harvest (°C)
- ***I*** – total hours of sun shining from second germination to hop harvest
- F3 – second germination after pruning
- F9 – technological maturity of hop cones (harvest time)
- *ET*_*0*(*VII*)_ – average daily reference crop evapotranspiration in July (mm day^-1^)

This equation, based on significant analytical data, unfortunately does not allow reliable prognosis of alpha-acids accumulation in technological maturity of hop cones.

However, nowadays because of very high hop supply, prognosis of alpha-acids accumulation become very important in order to estimate the commodities. Thus, the aim of this research is to find a simple mathematical model due to sum of effective temperatures and rainfall from second germination after spring pruning till the technological maturity of hop cones, in order to achieve reliable prognosis of alpha-acids accumulation in hop cv. Aurora.

## Materials and methods

Research was carried out on hop cv. Aurora planted in hop garden in Croatia, near the village of Gregurovec (close to Križevci), during the six vegetation years (2001 – 2006, Srečec et al.^2008^). The soil type of examined hop garden is an eutric pseudogley or eutric podzoluvisol. Content of physiological active phosphorus and potash, analysed by the AL-method, was medium. Average content of P_2_O_5_ and K_2_O during the all six experimental years was 19.1 and 15.4 mg per 100 grams of soil, respectively. Content of humus in soil was very low, only 1.48%. Fertilization was provided on the basis of plant uptake for phosphorus and potash and in three splits of nitrogen (50 + 70 + 50 kg/ha N on 20^th^ May, 10^th^ June and 5^th^ July) in all 6 experimental years. Meteorological data in Croatia were collected in Agro Meteorology Station in Križevci, placed five kilometres far away from the hop garden.

Hop cones were sampled from the same plants each year in the phenological phase of technological maturity.

The empiric mathematical equation achieved in Croatia was checked in year of 2012 at the same cultivar grown in completely different agro-ecological conditions in Žalec, Slovenia. P_2_O_5_ and K_2_O in the hop garden was 46.0 and 20.1 mg per 100 grams of soil, respectively. Content of humus in soil was 2.9%. The soil is middle heavy, young alluvial soil, poorly developed on sandy-gravelly deposits of two rivers.

Meteorological data in Slovenia for year 2012 were collected in Agro Meteorology Station at the Slovenian Institute of Hop Research and Brewing placed near by the hop garden.

Sum of effective temperatures, during the hop vegetation, for every experimental year, was calculated by the following equation (2):2

where:

D*min*T - daily minimal temperature,

D*max*T - daily maximal temperature and

5°C is minimal temperature required for beginning of hop vegetation.

The content of alpha-acids was determined by the method of lead conductance value of hops, powder and pellets prescribed by Analytica - EBC 7.4 (Anon. 1998) and dry matter content by the method of moisture content of hops and hop products prescribed by Analytica - EBC 7.2 (Anon. 1998). The boarder of repeatability (r_95_) for method of lead conductance value is 0.2 and the boarder for reproducibility (R_95_) is1.

During the examined vegetation years in Croatia, samples of hop cones were handpicked and analysed from the same 35 plants, randomly chosen and marked in the first research year of 2001 (Srečec et al. 2008) in the same hop garden, which means five control plots with seven plants per each plot within the same hop garden. On the other hand, samples of hop cones in Slovenia in year of 2012 were also handpicked, but sampled randomly from different plants in the same hop garden.

Analyses of achieved analytical results were provided by software Eureqa Formulize 0.96 Beta (Nutonian, Inc.). Eureqa Formulize is a scientific data mining software package that searches for mathematical patterns hidden in data. Formulize’s user interface is organized as a set of seven tabs that correspond to the normal workflow through the program, and user guide is organized around those tabs (see: http://www.nutonian.com). In mathematical analyses of experimental data sum of effective temperatures and total rainfall were treated as independent variables (marked as *x* and *w* variables) and content of alpha-acids (%) as dependent variable (*y* variable).

## Results and discussion

**Figure 1:**
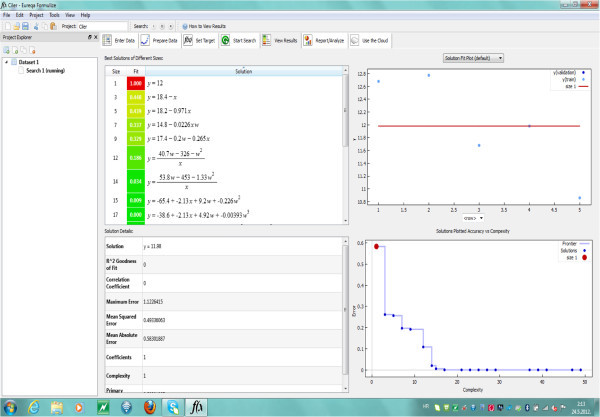
**Menu of Eurequa Formulize 0.96 on which is visible workflow (computing) with suggested equations and their error probabilities.**

After mathematical analyses of experimental data by Eureqa Formulize 0.96 Beta software 17 equations were offered (Figure 1).

After substituting the values of dependent and independent variables in all equations only one equation was chosen with p = 0.034 (p<0.05) (equation 3).3

Where:

*y* – alpha-acids content in dry matter (%)

*x* – sum of effective temperatures (°C) from second germination after the spring pruning till technological maturity

*w* – sum of total raifalls (mm) for the same period

However, that equation had to be refined by the authors, due to the negative results obtained and shifting the decimal point one space to the left (equation 4), because fraction must be divided by −10 in order to achieve reliable values.4

Reliability of equation (4) was checked by inserting the values for the dependent and independent variables for the ten-year period for Croatian agro-ecological conditions (Table 1).

**Table 1:** Reliability of mathematical model for accumulation of alpha-acids in hop cultivar Aurora in location of Gregurovec, Croatia during the six vegetation periods (2001-2006*)

Crop year	Sum of effective temperatures (°C)	Sum of total rainfalls (mm)	***ET***_***0***_in July	Calculated content of alpha-acids (%) in dry matter	Analysed content of alpha-acids (%) in dry matter (mean)	Difference (alpha calc. – alpha analysed)
2001	1698.4	393.7	4.26	10.9	11.6	0.7
2002	1932.1	425.8	4.20	11.3	11.1	0.2
2003	1994,4	175.2	5.5	2.5	6.7	- 4.2 (n/r)
2004	1856.7	398.5	4.28	10.2	10.0	0.2
2005	1920.8	403.3	4.38	10.1	9.7	0.5
2006	1872.1	382.0	4.42	9.2	9.3	0.1

* previous functional equation for accumulation of alpha acids based on the results of linear and multiple correlations between weather conditions and accumulation of alpha acids described by Srečec et al. 2008n/r – not reliable.

The results were tested also in agro-ecological conditions of Žalec, Slovenia during the 2012 (Table 2).

**Table 2:** **Reliability of mathematical model for accumulation of alpha-acids in hop cultivar Aurora in location of Žalec, Slovenia in 2012**

Crop year	Sum of effective temperatures (°C)	Sum of total rainfalls (mm)	***ET***_***0***_in July	Calculated content of alpha-acids (%) in dry matter (mean)	Detected content of alpha-acids (%) in dry matter (mean)	Difference (alpha calc. – alpha detect.)
2012	1766.2	400.4	4.2	10.8	10.2	0.6

It is obvious that the differences between calculated and detected content of alpha-acids varied from 0.1 to 0.7, which is within boarders of repeatability (r_95_ = 0.2) and reproducibility (R_95_ = 1) for EBC 7.4 method (Anon 1998). However, this equation is not reliable in extremely drought year, like the year of 2003 was. That confirms results of Srečec et al. (2008), who found a negative correlation, determined by Spearman’s rank correlation, during the phonological phase of hop cones formation, between average daily reference crop evapotranspiration (ET_0_) in July and yield of hop cones, r_*s*_ = − 0.75 (p < 0.05), as well as between ET_0_ and yield of alpha-acids in the same period, r_*s*_ = − 0.88 (p < 0.05). This is also possible to explain with results of Pavlovič et al. (2012), according to them, rainfall quantity from June 18 to July 22 shows the highest correlation with alpha-acid contents and impact of rainfall begins to decline after July 29. However, in time after July 29, the formation of glandular trichomes starts and the positive Spearman’s rank correlations were found between the average number of glandular trichomes and the content of alpha-acids (r_*s*_ = 0.90; p<0.05) and also between the average volume of glandular trichomes and content of alpha-acids (r_*s*_ = 0.97; p<0.05) (Srečec et al. 2011).

Finally, considering these results it is possible to suggest following general equation for alpha-acids accumulation in hop cv. Aurora (equation 5):5

In case of Aurora hop cultivar coefficients *k*_*1*_, *k*_*2*_ and *k*_*3*_ are determined, which have to be determined for the other hop cultivars.

## Conclusion

Achieved results confirms the results of Srečec et al. (2008) and using the Eureqa Formulize 0.96 Beta software allows reliable mathematical analyses but only if linear and multiple correlations of experimental data are previously provided. However, these results as well as results of previous authors show that weather conditions, during the hop vegetation have a stronger influence on accumulation of alpha-acids in technological maturity of hop cones than soil conditions.
